# Possible Down Regulation of the p16 Gene Promoter in Individuals with Hepatocellular Carcinoma

**DOI:** 10.5812/kowsar.1735143X.732

**Published:** 2011-09-01

**Authors:** Oranous Bashti Shiraz, Hamid Galehdari, Majid Yavarian, Bita Geramizadeh

**Affiliations:** 1 Department of Genetics, Faculty of Science, Chamran University, Ahwaz, IR Iran; 2 Cancer Research Center, Jondishapour Medical University, Ahwaz, IR Iran; 3 Hematology Research Center, Shiraz University of Medical Sciences, Shiraz, IR Iran; 4 Transplant Research Center, Department of Pathology, Shiraz University of Medical Sciences, Shiraz, IR Iran

**Keywords:** p16, Hepatocellular carcinoma, Bisulfite, Direct sequencing methylation

## Abstract

**Background:**

The p16 tumor suppressor gene is an important negative regulator of the cell cycle. Inactivation of p16, especially via promoter hypermethylation, has been found in numerous human cancers such as breast, lung, colorectal, and liver.

**Objectives:**

To determine the role of epigenetic methylation in p16 regulation in Iranian patients with hepatocellular carcinoma (HCC).

**Patients and Methods:**

The methylation pattern in the p16 gene promoter was analyzed by bisulfite direct sequencing in 43 paraffin-embedded formalin-fixed tissues from patients with HCC. In addition, normal specimens from liver graft donors were used as the control group.

**Results:**

The bisulfite direct sequencing showed heterozygous hypermethylation in 13.9% of individuals with HCC. Homozygous methylation within the GC-box IV was detected in another 58.1% of the patients.

**Conclusions:**

It is proposed that methylation, but not necessarily hypermethylation, may play a role in the down-regulation of the p16 gene promoter at least in some Iranian patients with HCC.

## 1. Background

**Figure 1:**
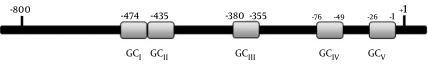
An 800-bp Portion of the Human p16 Gene Promoter Located Upstream of the Initiation Codon. The Promoter Region Contains 5 ConsensusGC Boxes, Which are Often the Targets for Methylation-Mediated Inactivationin Diverse Human Cancers, Including HCC.

Hepatocellular carcinoma (HCC), one of the most fatal human malignancies, is characterized by late presentation, fast progression, and limited response to therapy [1]. HCC is commonly associated with the chronic liver diseases caused by infection with the hepatitis B virus (HBV) and/or the hepatitis C virus (HCV), excessive alcohol consumption, aflatoxin, and certain metabolic diseases [2][3][4][5]. Inactivation of tumor suppressor genes and activation of oncogenes initiated by genetic and epigenetic differences may play an important role in carcinogenesis. The p16ink4a gene is a tumor suppressor that acts as a negative regulator of the cell cycle by binding to and inhibiting cyclin-dependent kinase 4 (CDK4) [6]. Reduced expression of the p16 gene results in uncontrolled division of cells. Several mechanisms that lead to p16 inactivation have been described, including point mutations, homozygous deletions, and promoter hypermethylation [7][8][9], and hypermethylation of the p16 gene promoter has been shown to occur more frequently in HCC patients [10][11][12]. The p16 gene promoter contains 5 GC boxes, which are termed GCI to GCV. The boxes cover a region located upstream of the translational start site from nucleotide -474 to -1 (Figure 1) [13].

## 2. Objectives

In the present study, we used direct bisulfite sequencing in order to detect the methylation patterns of GC box IV, GC box V, and a portion of exon 1 in the p16 gene promoter in Iranian patients with HCC.

## 3. Patients and Methods

### 3.1. DNA Extraction

Paraffin-embedded formalin-fixed (PEFF) tissues from 43 patients with HCC were collected from Namazi hospital (Shiraz, Iran) between September 2005 and December 2009. For the controls, 20 normal liver tissue samples were obtained from volunteer liver graft donors. The donors were brain dead, and their families allowed their organs to be donated. Sections (10 μm) were cut from the PEFF tissue blocks and were deparaffinized with xylene. Genomic DNA was extracted using a DNeasy Blood and Tissue Kit according to the manufacturer's instructions (Qiagen, Valencia, CA, USA).

### 3.2. Bisulfite Modification

Bisulfite modification was performed based on the principle that bisulfite converts unmethylated cytosine residues to uracil, whereas methylated cytosine residues remain unaffected. Therefore, after bisulfite conversion, methylated and unmethylated cytosines were determined by direct sequencing. Bisulfite treatment of DNA was performed according to the instructions in the EpiTect Bisulfite Kit (Qiagen).

### 3.3. Bisulfite Direct Sequencing

In the bisulfite direct sequencing method, primers should be designed to amplify both methylated and unmethylated sequences. In addition, they should not contain CpG- cytosines, because they are not complementary to methylated cytosines, which are not affected by sodium bisulfite. Finally, after direct sequencing, all sites with unmethylated cytosines are displayed as thymines in the amplified sense strand and as adenines in the amplified antisense strand. A 191-basepair fragment in the p16 gene promoter, including 19 CpG dinucleotides, was amplified by nested polymerase chain reaction (PCR). The first round of amplification was performed with 100 ng of bisulfite-treated DNA. The primers for the first PCR were 5'-TTTTTAGAGGATTTGAGGGATAGG-3' (forward) and 5'-CTACCTAATTCCAATTCCCCTACAAACTTC-3' (reverse). The initial PCR conditions were as follows: 94°C for 1 min; 5 cycles at 94°C for 45 s, 65°C for 45 s, and 72°C for 30 s; 5 cycles at 94°C for 45 s, 64°C for 45 s, and 72°C for 30 s; and then 25 cycles at 94°C for 45 s, 63°C for 45 s, and 72°C for 30 s, with final extension step at 72°C for 5 min. An aliquot of the PCR product was used as the template for the second (nested) PCR. The nested PCR was performed using 5'-AGAAAGAGGAGGGGTTGGTTGG-3' as the forward primer and 5'-ACRCCCRCACCTCCTCTACC-3' as the reverse primer. Nested PCR was performed with an initial denaturing step at 94°C for 1 min, followed by 35 cycles at 94°C for 45 s, 61°C for 45 s, and 72°C for 30 s. The subsequent cycle sequencing reaction was performed using an automatic sequencer (ABI Company).

### 3.4. Statistical Analysis

The correlation between the clinicopathological pa rameters and p16 methylation was analyzed using the Chi-square test and Student's t-test. A P < 0.05 was considered statistically significant.

## 4. Results

A total of 43 HCC patients were analyzed according to age, sex, cirrhosis, and hepatitis and tumor pathological grade. (Table 1) Among the affected individuals, the sex ratio was 28 males to 15 females, with an average age of 48 years. Other HCC-related risk factors were as follows: cirrhosis (34.8%), hepatitis B infection (9.3%), and hepatitis C infection (2.3%). The samples were examined pathologically and classified as well differentiated HCC (n = 24), moderately differentiated HCC (n = 14), and poorly differentiated HCC (n = 5).

**Figure 2:**
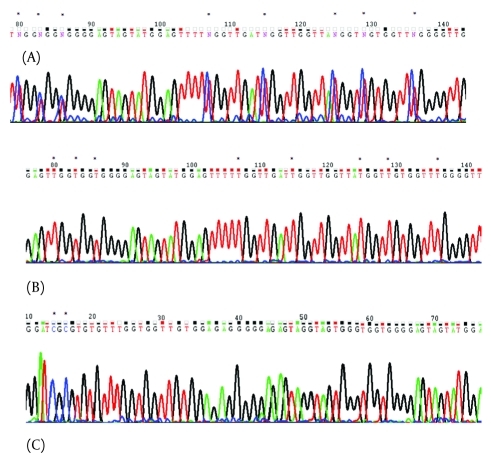
(A) A Portion of the p16 Gene Promoter that was Heterozygously Methylated in the HCC Samples; (B) the Same Region of the Promoter in Normal Tissues was Unmethylated. (C) A Portion of the p16 Promoter that was Methylated within GC-Box IV. Asterisks Indicate Methylated Nucleotides.

A 191-bp region of the p16 gene promoter in archival PEFF samples from 43 HCC cases and an additional 20 control samples prepared from normal liver tissue was subjected to bisulfite direct sequencing. We found heterozygous hypermethylation in 13.9% (n = 6) of the HCC samples, and homozygous methylation in GC-box IV was found in 58.1% (n = 25) of the HCC samples. In contrast, we did not observe any modification in the normal samples (Figure 2).

**Table 1:** Characteristics of HCC Patients and the Control Group

	** HCC **a** Patients **	** Normal Controls **
Number of cases	43	20
Gender		
Males	28	14
Females	15	6
Average age, y	48	45
Cirrhosis	15	
Hepatitis B	4	
Hepatitis C	1	
Grade		
Wd a	24	
Md a	14	
Pd a	5	

^a^ Abbreviations: HCC, hepatocellular carcinoma; Md, moderately differentiated;Pd, poorly differentiated HCC; Wd, well differentiated

The clinical characteristics of patients who were positive for hypermethylation and GC-box IV methylation are listed in Table 2. No significant correlation was found between abnormal methylation in HCC and age, gender, or tumor grade.

**Table 2:** Characteristics of Patients and Methylation and Hypermethylation of the p16 Gene Promoter of HCC Patients and the Control Group

	**No.(n=43)**	**p16 Methylation/ hypermethylation (+)**	**p16 Methylation/ hypermethylation (-)**	**Chi-Square**	**P value**
Gender				0.02	0.05
Male	28	20	8		
Female	15	11	4		
Age, y					
6–80	43	49.89 ± 5.22 a	45.67 ± 15.23 a	0.46 b	≥ 0.05
Cirrhosis				0.99	> 0.05
Affected	15	8	7		
Not affected	28	19	9		
Hepatitis B				0^c^	0^c^
Affected	4	1	3		
Not affected	39	30	9		
Hepatitis C				0^c^	0^c^
Affected	1	0	1		
Not affected	42	31	11		
Grade				0.84	>0.05
Wd d	24	16	8		
Md d	14	11	3		
Pd d	5	4	2		

^a^ Mean ± SD

^b^ Student’s t-test

^c^ Because of the low number of samples, it was not possible to calculate a P value.

^d^ Abbreviations: Md, moderately differentiated; Pd, poorly differentiated HCC; Wd, well differentiated

## 5. Discussion

DNA methylation is a type of chemical DNA modification in which a methyl group is added to carbon 5 of the cytosine ring. This reaction is carried out by a group of enzymes called DNA methyltransferases, which regulate gene transcription through DNA methylation [14]. Interest in the field of DNA methylation has increased significantly in recent years because of its major role in the cancer process [15][16]. Aberrant methylation patterns in tumors include global hypomethylation and localized hypermethylation at CpG islands; however, these 2 types of epigenetic abnormalities usually seem to affect different DNA sequences. In most cancer types, genomic hypomethylation usually appears in repeated DNA sequences, while hypermethylation is most often observed within the CpG islands in the promoter regions of genes, frequently tumor suppressor genes [17]. In this context, p16 acts as a negative regulator of the cell cycle, and hypermethylation in the p16 promoter region has been reported to occur frequently in several human cancers such as HCC [18][19][20]. In contrast, other studies have shown a low frequency of hypermethylation within the p16 gene in individuals with HCC [21].

In the current study, the methylation state of the p16 gene promoter was analyzed by bisulfite direct sequencing in Iranian patients with HCC. In 13.9% (n = 6) of samples, heterozygous hypermethylation was detected within the p16 gene promoter. Furthermore, DNA methylation was observed within GC-box IV in 58.1% (n = 25) of tumor tissue samples. All normal samples were negative for every type of methylation tested. Therefore, we reason that methylation in GC-box IV may sufficiently decrease the transcriptional activity of the p16 gene. Several reports have suggested that methylation of at least 1 cytosine would significantly down-regulate p16 promoter activity [22]. In general, RNA helicase A interacts with the regulatory region of genes and facilitates transcription activity, such that methylation would significantly alter the interaction between the enzyme and the substrate [23]. On the basis of the abovementioned negative results in the normal specimens, we propose that methylation, but not necessarily hypermethylation, plays a role in the down-regulation of the p16 gene promoter at least in Iranian individuals with HCC.

The methylation results have been compared to the clinicopathological features. It appears that cirrhosis is the most common risk factor for the development of HCC in our patients. Cirrhosis is a progressive disease that often leads to cancer [24]. The frequency of hepatitis B (1.7-5%) and hepatitis C (0.5-1%) infection and aflatoxin contamination could also explain the relatively high frequency of this disease in Iran [25]. Among our patients, 6 were under the age of 18 years; 2 of them were affected with tyrosinemia type 1, and 1 of them had Fanconi anemia. All of the abovementioned factors and diseases have a negative influence on liver function and thus increase the probability of HCC. For the other 3 young patients, there is a possibility that they inherited an affected gene, in particular a tumor suppressor gene. According to the 2 hit hypotheses, when an affected allele is inherited, the probability of second allele inactivation via deletion (LOH), mutation, and/or promoter hypermethylation increases [26]. Statistical analysis also revealed no significant correlation between the presence of abnormal methylation in the affected tissues and gender, age, and cirrhosis. Comparing p16 methylation and hypermethylation in the HCC tissues according to tumor grade, methylation or hypermethylation was detected in 16 of 24 (61%) well differentiated HCCs, 11 of 14 (78%) moderately differentiated HCCs, and 4 of 5 (80%) poorly differentiated HCCs. Therefore, there was no association between methylation or hypermethylation and tumor grade. This indicates that methylation has a negative effect on gene expression, both in the primary stages of tumor formation and during tumor progression. Finally, we assume that other mechanisms, such as LOH and/or point mutations, might be factors responsible for tumor suppressor gene loss of function. However, additional evaluation is needed to understand the role of the epigenetic factors that influence p16, in particular, and the onset of HCC in Iranian individuals in general.
